# Massive bioconstructions built by *Neopycnodonte cochlear* (Mollusca, Bivalvia) in a mesophotic environment in the central Mediterranean Sea

**DOI:** 10.1038/s41598-020-63241-y

**Published:** 2020-04-14

**Authors:** Frine Cardone, Giuseppe Corriero, Caterina Longo, Maria Mercurio, Senem Onen Tarantini, Maria Flavia Gravina, Stefania Lisco, Massimo Moretti, Francesco De Giosa, Adriana Giangrande, Carlotta Nonnis Marzano, Cataldo Pierri

**Affiliations:** 1 Dipartimento di Biologia, Università degli Studi di Bari Aldo Moro, Via Orabona 4 - 70125, Bari, Italy; 2 Consorzio Nazionale Interuniversitario per le Scienze del Mare (CoNISMa), Piazzale Flaminio 9 - 00196, Roma, Italy; 3 Istituto di Ricerca sugli Ecosistemi Terrestri (CNR-IRET), Via Salaria km 29.300 - 00015 Monterotondo Scalo, Roma, Italy; 4 Dipartimento di Biologia, Università di Roma “Tor Vergata”, Via della Ricerca Scientifica s.n.c. 00133, Roma, Italy; 5 Dipartimento di Scienze della Terra e Geoambientali, Università degli Studi di Bari Aldo Moro, Via Orabona 4 - 70125, Bari, Italy; 6 Environmental Surveys S.r.l. (ENSU), Via de Gasperi - 74123, Taranto, Italy; 7 Dipartimento di Scienze e Tecnologie Biologiche ed Ambientali, Università del Salento, Via Provinciale Lecce-Monteroni - 73100, Lecce, Italy

**Keywords:** Ecology, Zoology

## Abstract

The present paper provides a multidisciplinary fine-scale description of a Mediterranean mesophotic new habitat dominated by the bivalve *Neopycnodonte cochlear* (Poli, 1795), building large and thick pinnacles on vertical cliffs at two study areas along the southern Italian coast. The pinnacles, constituted by a multilayered aggregation of living and dead specimens of *N. cochlear*, were interconnected with each other to form a framework of high structural complexity, never observed before for this species. The bioconstruction, considerably extended, resulted very complex and diversified in the associated community of structuring organisms. This latter included 165 taxa attributable to different ecological groups occurring in different microhabitats of the bioconstruction. Among the secondary structuring taxa there were scleractinians, serpulids and bryozoans, all contributing to the deposition of calcium carbonate, and poriferans, helping to bind shells together or eroding carbonate by boring species. In comparison with coralligenous *sensu stricto* and the recently described Mediterranean mesophotic coral reef, the *Neopycnodonte* bioconstruction showed peculiar features, since it lacked the major contribution of encrusting coralline algae and scleractinians as reef builders, respectively.

## Introduction

The main marine bioconstruction in the Mediterranean Sea is localized in the euphotic zone and is well known under the name of coralligenous which is typically considered to be the climax biocoenosis of the circalittoral zone^1^. Coralligenous reefs are widely distributed and consist of thick carbonate concretions mainly built by red calcareous algae, with the variable contributions of sessile invertebrate calcium carbonate depositors (e.g., scleractinians, serpulids, bryozoans)^2–4^. The large amount of different habitats associated with such bioconstructions support the highest values of biodiversity in the Mediterranean Sea^2^. However, with increasing depth and as a result of light attenuation, benthic sessile invertebrates progressively replace algal concretions, becoming the most important habitat builders^5^.

The biogenic role of animal bioconstructors has been repeatedly studied in Mediterranean deep-water habitats, where the predominant colonial scleractinians build large three-dimensional (3D) carbonate structures referred to as Cold-Water Corals (CWC) and provide substrate and habitat for a multitude of other organisms^6–11^. Conversely, little attention has been given to the Mediterranean mesophotic environment that, receiving less than 3% of the surface irradiance, represents the transitional zone between euphotic and dark environments.

Bioconstructions of the mesophotic habitat, well known in tropical waters^12–15^, have only recently been investigated in the Mediterranean area. An important contribution to these carbonate structures seems to be provided by the bivalve *Neopycnodonte cochlear*, which makes mass aggregations on the sea bottom on both soft and hard substrates, supporting the development of a rich benthic fauna^16–18^. In addition, the role of zoobenthic taxa as builders in Mediterranean mesophotic environments has been emphasized in a recent paper^5^ describing an outstanding carbonate bioconstruction built mainly by scleractinians along the southern Italian Adriatic coast.

Available studies reveal that Mediterranean mesophotic bioconstructions can represent notable biodiversity hotspots^5,19–21^ and include species of considerable economic and ecological importance^20,22^. Moreover, as a result of their vulnerability, habitats associated with mesophotic bioconstructions are protected by international agreements^23^. Data in the literature, however, are mainly based on Remotely Operated Vehicles (ROV) observations and mostly focus on a few conspicuous megabenthic species^24–27^. Thus, the main morphological features of these bioconstructions remain widely unknown, as well as their biological diversity, both in terms of structuring builder species and the associated fauna.

The aim of the present study was to improve knowledge on mesophotic bioconstructions in the central Mediterranean Sea using a multiscale approach coupling marine biology and geology methods. In particular, the focus was on the characterization of the mesophotic habitat dominated by the bivalve *N. cochlear* along the southern Italian coast (northern Ionian Sea). The fine structure of these carbonate bioconstructions was for the first time investigated by describing their morphological framework and characterizing the structuring taxa associated with bioconstruction at two different sites. Our general goal was to highlight the role of *N. cochlear* as a peculiar ecosystem engineer in the mesophotic environment, contributing to a better understanding of the ecological role of mesophotic bioconstructions and enhancing the possible future application of effective management and conservation tools.

## Results

### Seafloor mapping

**Figure 1:**
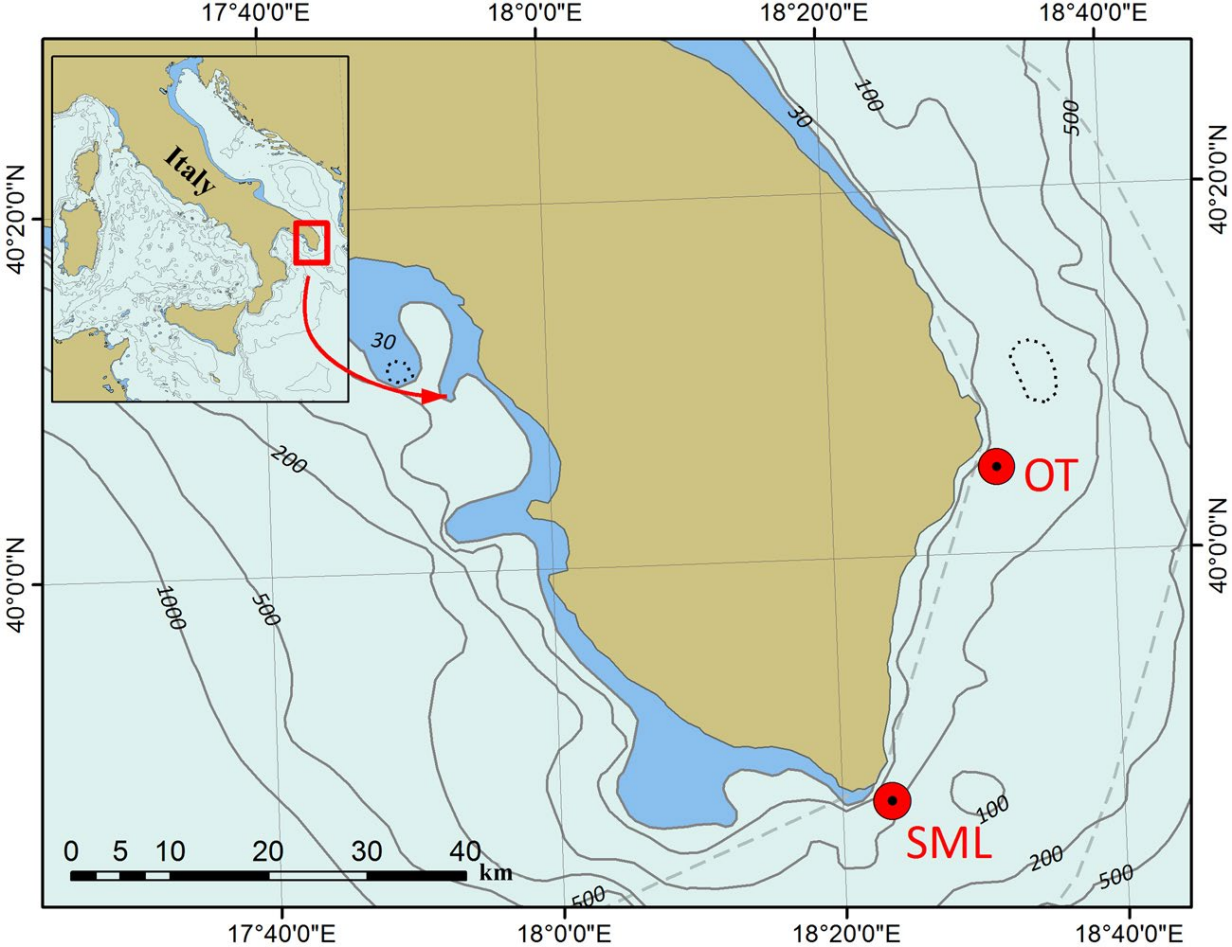
Locations of the two study areas: Otranto (OT) and Santa Maria di Leuca (SML). They occur off the southern Italian coast and are identified by red circles. Map has been created with ESRI ARCMAP 10.2, available at https://support.esri.com/en/products/desktop/arcgis-desktop/arcmap/10–2–2.

**Figure 2:**
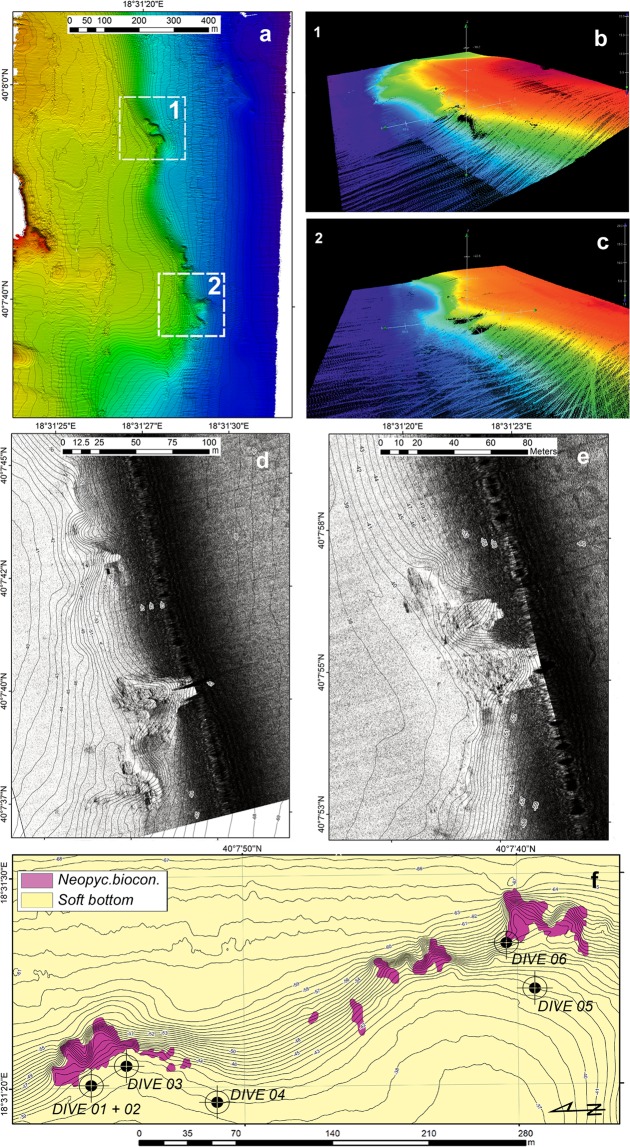
Geophysical survey of the Otranto area and localization of dive points. **(a)** Digital elevation model of the seafloor derived by the multibeam survey. (**b**,**c**) Areas 1 and 2 are characterized by the largest slope gradient. (**d**,**e**) Details of the raw data from the Side-Scan Sonar survey in areas 1 and 2. Note the presence of hard substrate along the slope. (**f**) Classi**f**ication of the seafloor and localization of dive points (black circles). Maps have been created with: (**a**) ESRI ARCMAP 10.2 + DTM and image produced by CARIS HIPS 9; (**b**,**c**) CARIS HIPS 9 (Subset editor); (**d**,**e**) ESRI ARCMAP 10.2 + SSS mosaics produced by CARIS SIPS 9; (**f**) ESRI ARCMAP 10.2, all available at https://support.esri.com/en/products/desktop/arcgis-desktop/arcmap/10–2–2 and https://www.teledynecaris.com/en/products/hips-and-sips/.

**Figure 3:**
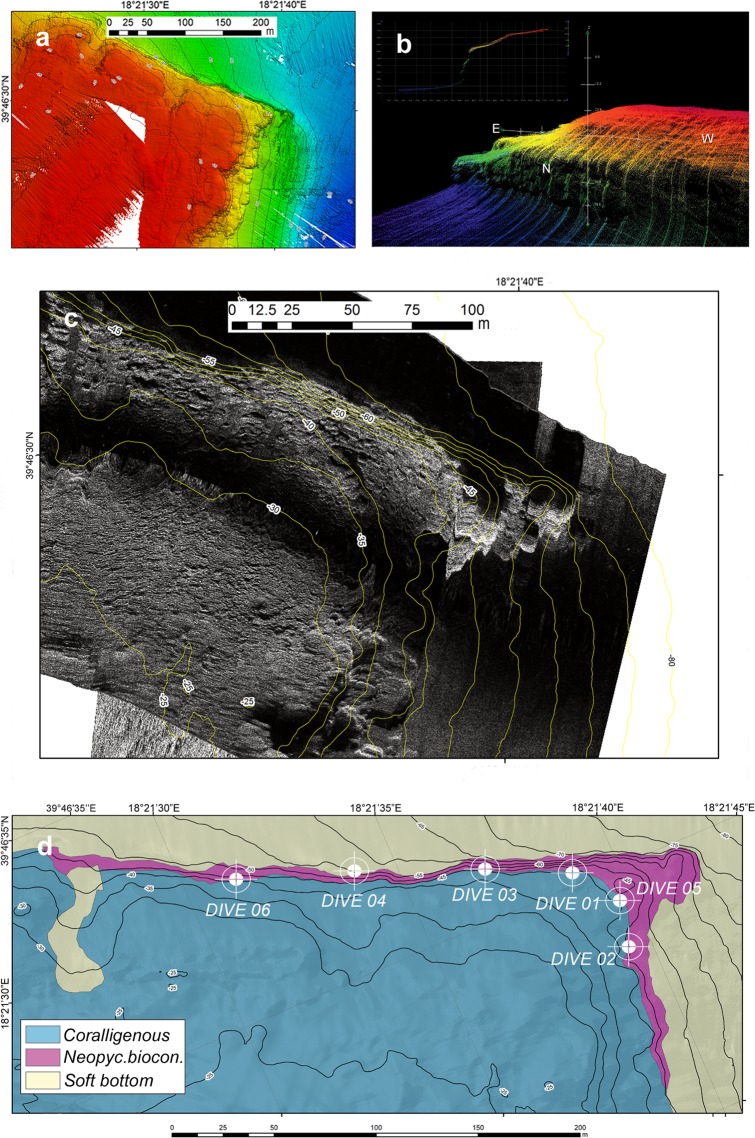
Geophysical survey of the Santa Maria di Leuca area and localization of dive points. **(a)** Digital elevation model of the seafloor derived by the multibeam survey. **(b)** Details of the ESE-WNW-oriented slope. Note that the slope seems to be very irregular along the portion with a higher gradient. **(c)** Raw data from the Side-Scan Sonar survey. **(d)** Classification of the seafloor and localization of dive points (white circles); the bioconstructions can be easily mapped, while shallower and deeper sectors seem to have a similar, even though unclear, acoustic signal. Maps have been created with: (**a**) ESRI ARCMAP 10.2 + DTM and image produced by CARIS HIPS 9; (**b**) CARIS HIPS 9 (Subset editor); (**c**) ESRI ARCMAP 10.2 + SSS mosaics produced by CARIS SIPS 9; (**d**) ESRI ARCMAP 10.2, all available at https://support.esri.com/en/products/desktop/arcgis-desktop/arcmap/10–2–2 and https://www.teledynecaris.com/en/products/hips-and-sips/.

The *Neopycnodonte* bioconstructions were studied in two different areas of the southern Apulian coast, Otranto (OT) and Santa Maria di Leuca (SML) (Fig. 1). In the OT area, *Neopycnodonte* bioconstructions were discontinuously detected along 600 m of the coastline within a bathymetric range of 45–64 m, reaching a total length of 200 m (Fig. 2). In the SML area, a carbonate formation built by the mollusc bivalve almost uniformly covered the northern and eastern sides of the cliff for a total length of approximately 450 m in the bathymetric range of 45–70 m (Fig. 3).

The two study areas, OT and SML, showed similar geomorphological features (Figs. 2 and 3). The seafloor geometry was dominated by the presence of a slope that connected a large coastal flat area to deeper sectors (depth range: 39–64 m in OT and 27–70 m in SML). This slope ran parallel to the coastline in the OT area (NNW-SSE, Fig. 2a), while in the SML area, it ran along an ESE-WNW direction, transversally to the coastline (Fig. 3a). In both cases, the slope was locally steep and showed irregular morphology (Figs. 2b,c and 3b).

Data from the Side-Scan Sonar (SSS) survey allowed a more detailed assessment of the nature of the seafloor. In the OT area, the acoustic signal was monotonous in the shallower (39 m) and deeper sectors (64 m), likely in relation to the presence of soft bottom (Fig. 2d,e). Rocky substrate randomly occurred in only the slope sectors, forming localized submerged “headlands” with an irregular slope and a nearly flat top.

In the SML area, the flat and shallow sectors (25–27 m) had a rocky substrate (Fig. 3c). The mosaicked acoustic signal suggested the presence of coralligenous bioconstructions or concretions, while close to the top of the slope (30 m), the substrate showed acoustic features that can be interpreted as small bioconstructions with irregular morphology (Fig. 3c,d). The slope was stiff and continuous, showing a complex acoustic signal likely related to the presence of large-scale bioconstructions (comparable with the irregular slope of the multibeam dataset (Fig. 3b). Deeper areas (70 m) seemed to have a fine-grained sediment substrate.

### Structure of the bioconstructions: building and structuring taxa

**Figure 4:**
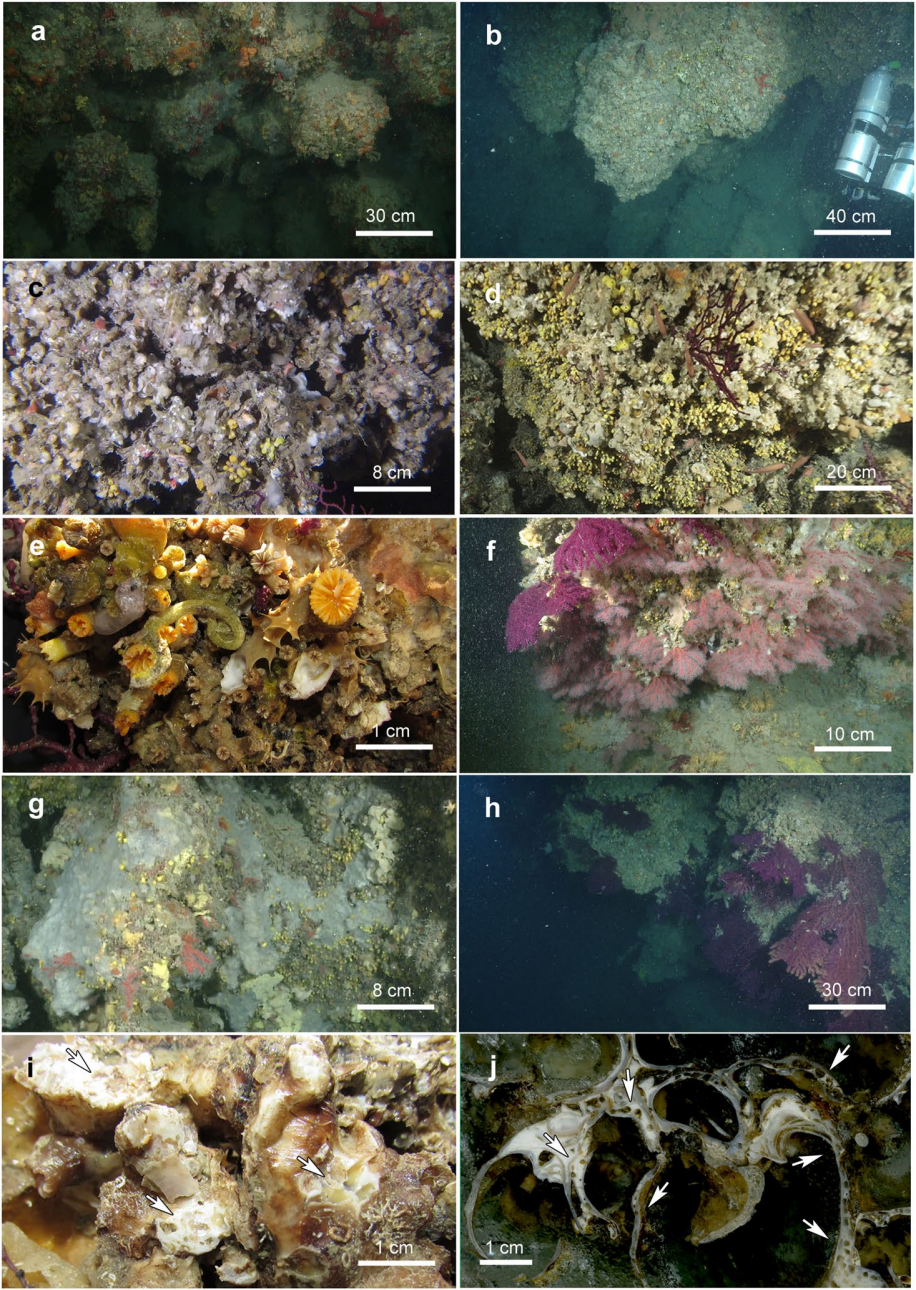
The *Neopycnodonte* bioconstructions. Underwater images of the bioconstructions: (**a**) globose formations at Otranto and **(b)** thick pinnacles at Santa Maria di Leuca protruding perpendicularly with respect to the cliff and interconnected with one another to form a framework of high structural complexity. **(c)** Detail of the *Neopycnodonte* bioconstruction formed by the massive, multilayered aggregation of shells. **(d, e)** Scleractinian facies**. (e)** Detail showing the main structuring taxa: *Cladopsammia rolandi/Leptopsammia pruvoti* complex (yellow corallites) and *Hoplangia durotrix* (light brown corallites). **(f)** *Corallium rubrum* facies. **(g)** Large portion of the substrate covered by the encrusting sponge *Dendroxea lenis* (grey). **(h)** *Paramuricea clavata* facies characterized by large colonies. **(i, j)** Detail of *Neopycnodonte* bioconstruction heavily infested by the boring sponge *Siphonodictyon infestum* (arrows) in a fresh sample **(i)** and in a sample embedded in resin **(j)**.

**Figure 5:**
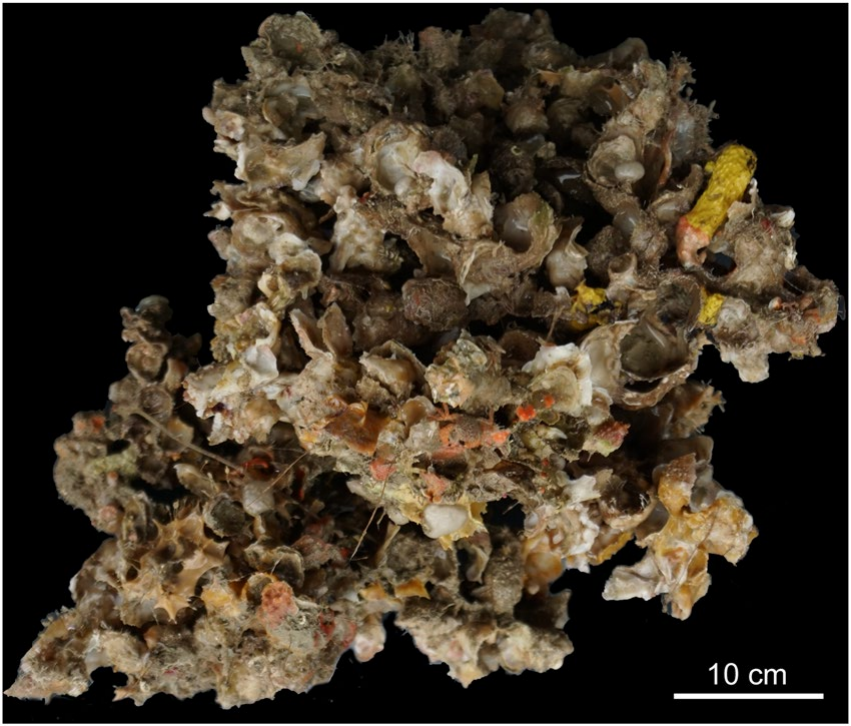
Large sample of the bioconstruction after collection. *Neopycnodonte* shells mainly appear irregularly arranged, forming a framework of high structural complexity.

**Figure 6:**
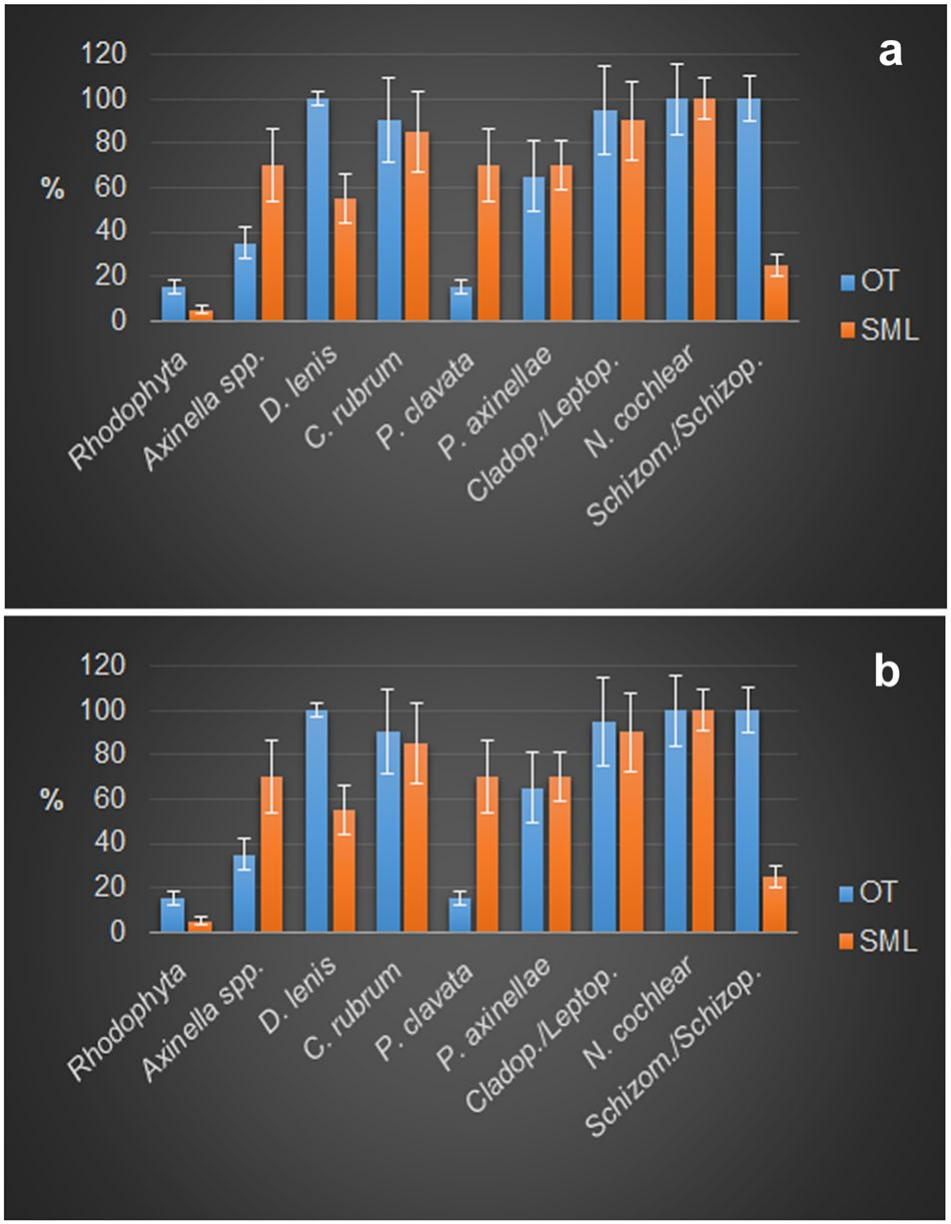
Contribution of the different structuring taxa to the bioconstruction  at Otranto (OT) and Santa Maria di Leuca (SML). **(a)** Frequency and **(b)** covering values of the main structuring taxa (mean %  ± s.e.). (*D. = Dendroxea; C. = Corallium; P. = Paramuricea; P. = Parazoanthus; Cladop*./*Leptop*. = *Cladopsammia*/*Leptopsammia; N. = Neopycnodonte; Schizom./Schizop. = Schizomavella/Schizoporella*).

**Figure 7:**
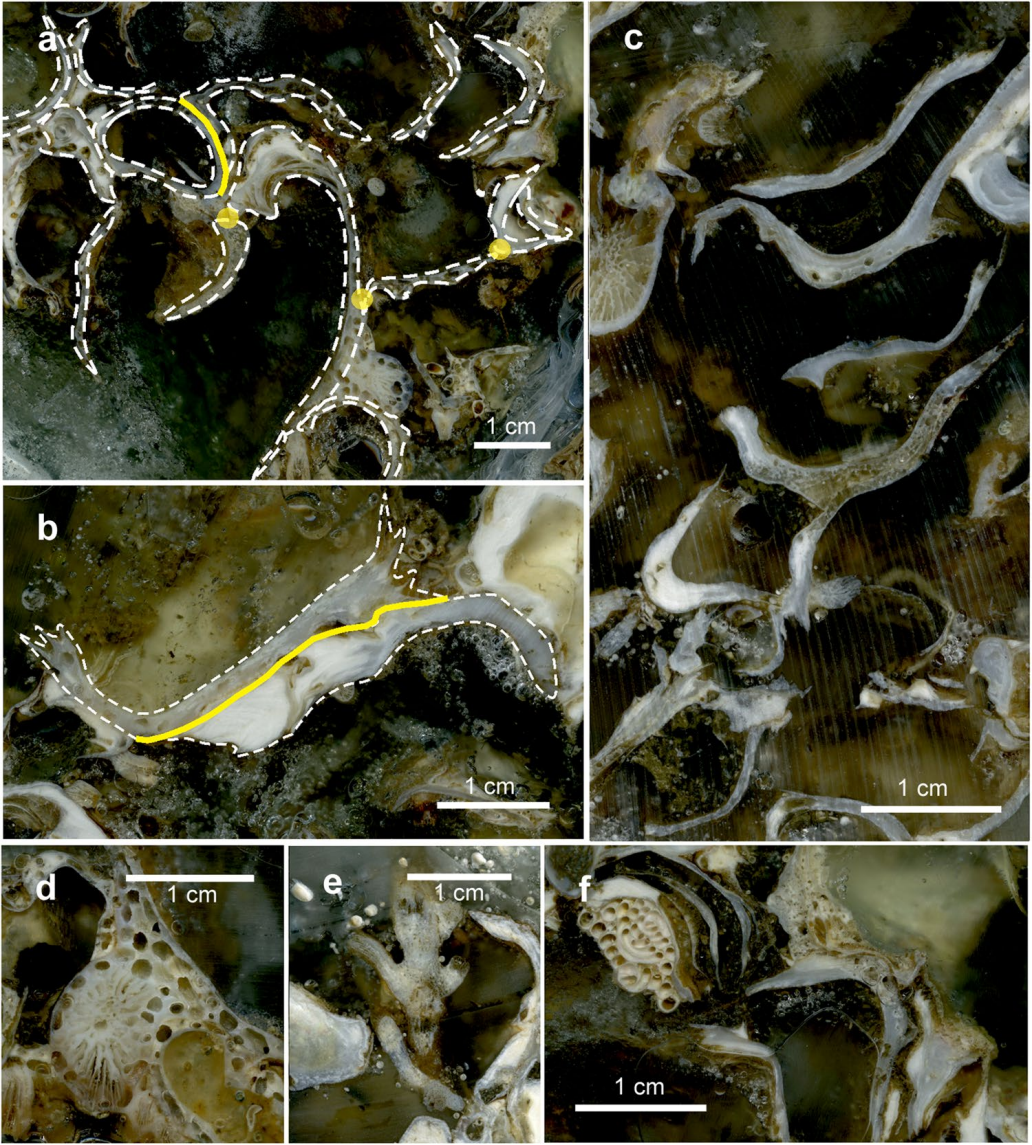
Details of the large-scale slices of the bioconstruction impregnated with epoxy resin. **(a)** Shells sharing single points (yellow circles) or large surfaces (yellow line). **(b)** Parallel *Neopycnodonte* shells that grow together, sharing large surfaces (yellow line). **(c)** Locally, the bioconstruction shows a remarkable porosity. **(d)** Traces of sponge perforations. **(e)** Scleractinian corallites on a mollusc shell. **(f)** Serpulids and bryozoans encrusting the surface of *Neopycnodonte* shells.

In both study areas, the bioconstructions developed in thick pinnacles or globose formations, protruding perpendicularly with respect to the cliff for approximately 50 cm at OT and for more than 1.5 m at SML and often interconnected with one another to form a framework of high structural complexity (Fig. 4a,b). The pinnacles were organized in successive terraces proceeding from the top to the bottom of the bioconstruction (see Supplementary Videos 1 and 2). According to the analysis of the video images, sampled material and resin slices, the pinnacles and their basal layer were mainly formed by the massive, multilayered aggregation of shells of *Neopycnodonte cochlear* (Poli, 1795) (Figs. 4c and 5). In both study areas, *N*. *cochlear* occurred in 100% of the analysed images, with average covering values of 84 and 82% in OT and SML, respectively (Fig. 6). At both sites, most of the bioconstructions were composed of dead specimens of *N. cochlear*. Live specimens were present in scattered clusters of a few individuals (6–20) grafted onto the superficial layer of the bioconstruction. Specifically, the large-scale analysis of the resin slices showed how the general framework of the bioconstruction was always shell supported and derived from the complex superposition of new valves on the preceding ones (Fig. 7). In general, *N. cochlear* specimens seemed to be irregularly arranged with respect to each other (Fig. 5), with individuals settling on the surface of older shells and sharing few points of contact with adjacent valves (Fig. 7a). Locally, some shells were arranged parallel to each other, thus increasing the contact surface between adjacent valves (Fig. 7b,c). The random orientation of the shells and the presence of point-like contacts determined the formation of an overall porous structure. At the same time, the contacts between larger surfaces favored the stability of the bioconstruction.

Among the other taxa structuring the bioconstructions, there were cnidarians, serpulids and bryozoans, all contributing to the deposition of calcium carbonate, and poriferans, helping to bind shells together or eroding carbonate by boring species. Indeed, boring sponges were often recorded living into the carbonate structures (Figs. 4i,j and 7d), and colonial scleractinians and serpulids were found within the concretion (Fig. 7e,f). In particular, among the secondary structuring taxa, the scleractinians *Cladopsammia rolandi* Lacaze-Duthiers, 1897, *Leptopsammia pruvoti* Lacaze-Duthiers, 1897, and, to a lesser extent, *Caryophyllia* (*Caryophyllia) inornata* (Duncan, 1878) and *Hoplangia durotrix* Gosse, 1860, were the most frequent structuring species (Fig. 4d,e and 6), having a constant presence across the bioconstructions. The alcyonacean *Corallium rubrum* (Linnaeus, 1758) also strongly contributed to the bioconstructions (Fig. 6). It showed a patchy distribution, with aggregates of several specimens concentrated below the pinnacles of the structure (Fig. 4f).

The outer portions of the bioconstructions as well as the reef interstices were extensively encrusted by serpulid tubes. In particular, most spirorbid polychaetes, especially *Protolaeospira* (*Protolaeospira*) *striata* (Quiévreux, 1963), *Pileolaria militaris* Claparède, 1870 and *Vinearia koehleri* (Caullery & Mesnil, 1897), colonized the bare parts of the substrate, such as the external edges of the shell of living *Neopycnodonte* specimens and their smooth inner parts, corresponding to the pioneering role that these organisms played in the community colonization pattern. Other spirorbid species exhibited their particular adaptation to the cryptic and dark crevices of the bioconstruction according to their small dimensions and often-wrapped tubes.

The bryozoans *Schizomavella* spp. and *Schizoporella* spp., particularly well represented in the OT study area (Fig. 6), formed thin crusts on the reef surface that contributed to the compactness of the structure. Sponges were mainly represented by encrusting species covering large portions of substrate. Among them, *Dendroxea lenis* (Topsent, 1892) occurred frequently in both study areas, with covering values that reached 42% of the substrate of the bioconstruction (Figs. 4g and 6). In contrast, massive and erect forms were less represented in both study areas, with the exception of small specimens of *Axinella* spp. (Fig. 6), which were mainly concentrated in the sub-horizontal portions of the substrate. Among the boring sponges, *Siphonodictyon infestum* (Johnson, 1889*)* played an important role as a bioeroder of the bioconstruction (Fig. 4i,j).

In addition, the reef’s crevices were also inhabited by *Hiatella* spp. molluscs living as nestlers or as borers, enlarging the reef’s holes. Additionally, the soft-bottom bivalve *Kellia suborbicularis* (Montagu, 1803) preferred the sediment trapped in the crevices and the spaces among the *Neopycnodonte* shells. Finally, red coralline algae were only sporadically detectable during the analysis of both images and biological samples (Fig. 6).

Regarding large epibenthic taxa, dense populations of the gorgonians *Paramuricea clavata* (Risso, 1826*)* and *Eunicella cavolini* (Koch, 1887) characterized the seascapes of both the habitats at both sites, representing the main 3D habitat makers, although at OT gorgonians were limited to a few areas of the bioconstruction (Fig. 4h and 6).

**Figure 8:**
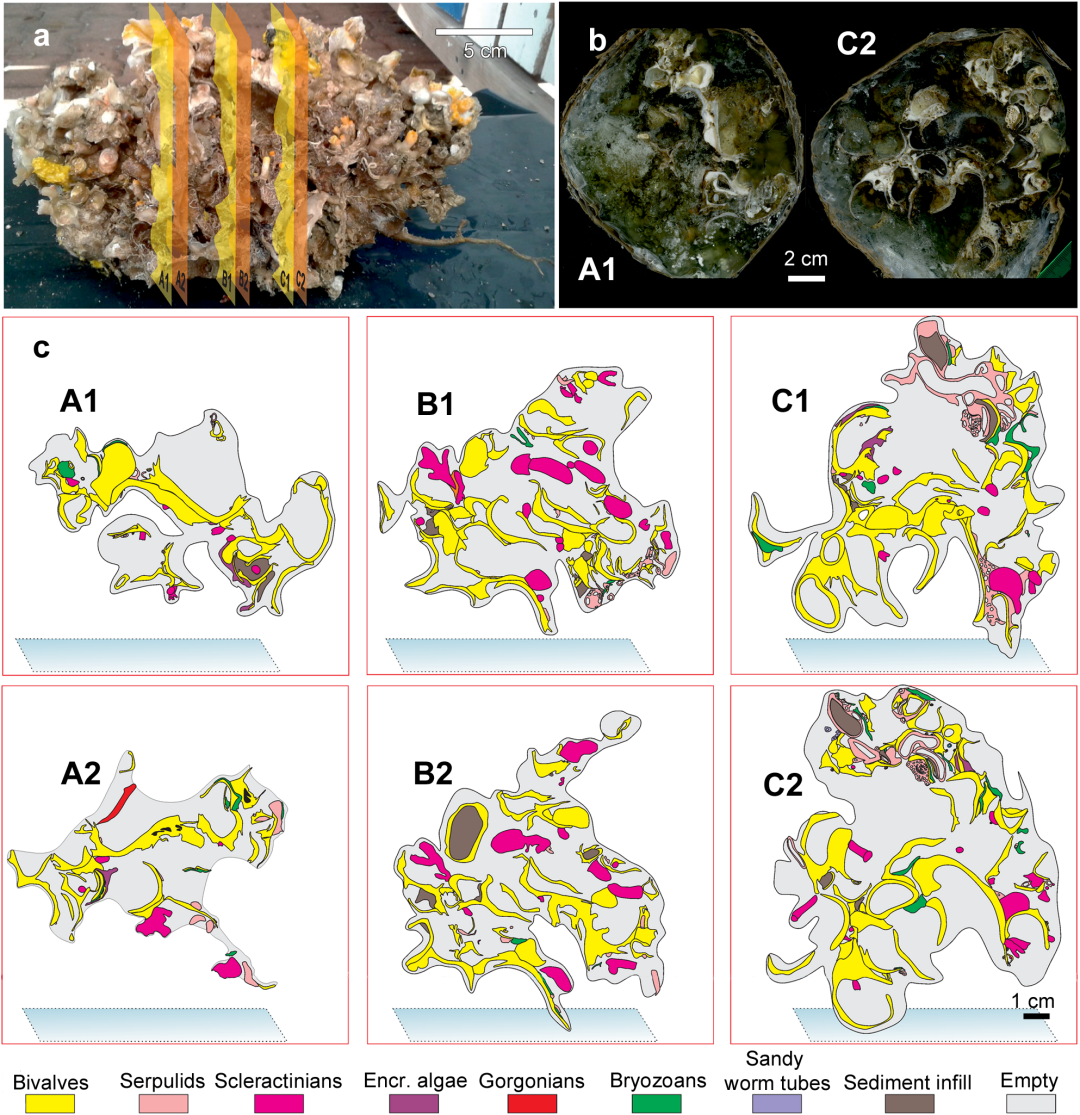
Bioconstruction block with details of the main structuring taxa identified. **(a)** Sampled block with indications of cut planes (yellow and orange polygons). **(b)** Examples of high-resolution images of the large-scale slices of the bioconstruction. **(c)** Compositional map of the different taxa.

**Figure 9:**
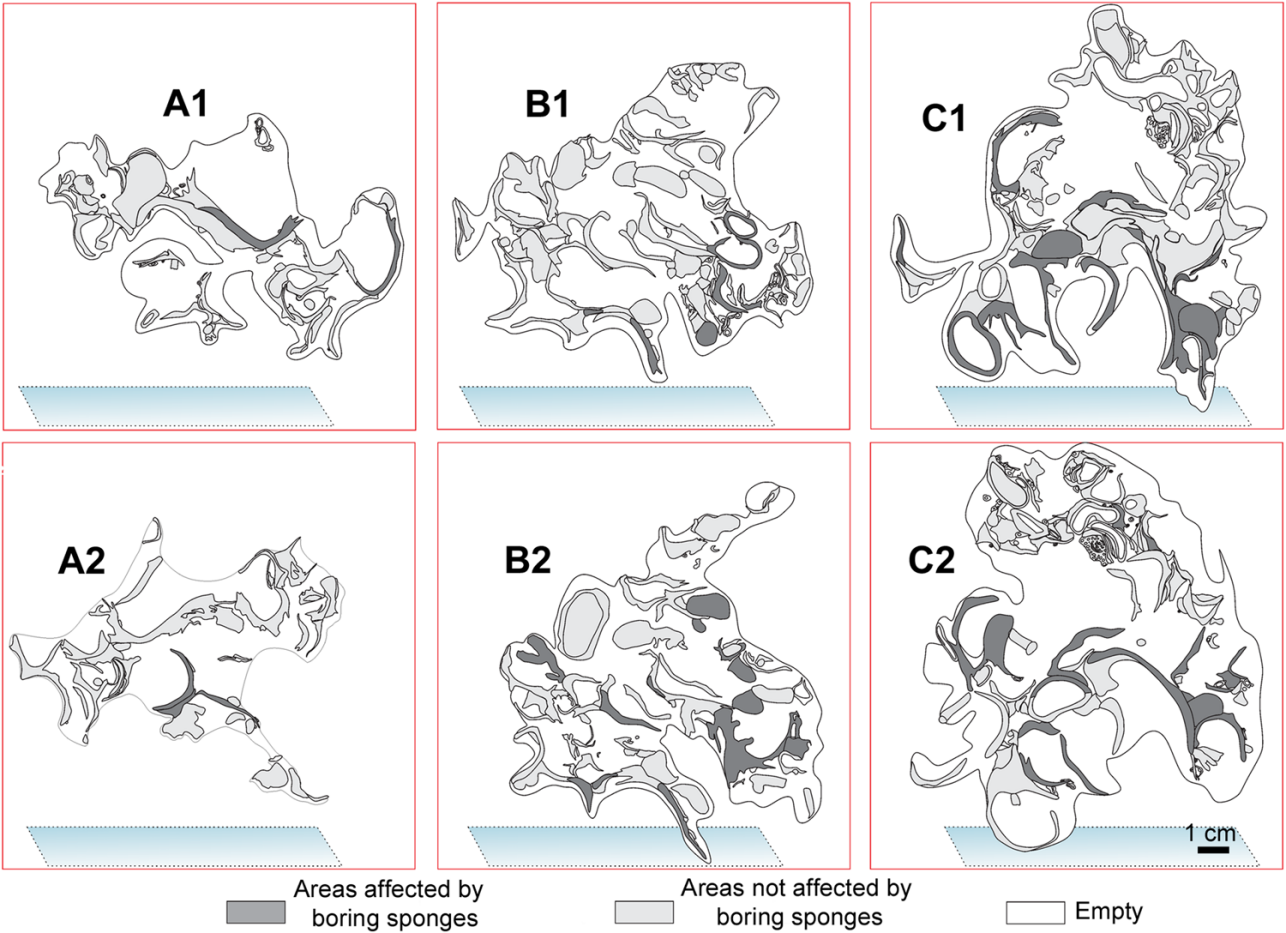
Distribution of the perforations produced by boring sponges in the same slices of Fig. 8.

The mapping of the area occupied by different taxa in the large-scale slices of the bioconstruction (Fig. 8) showed the following average percentage values: *Neopycnodonte* shells: 73.8 ± 7.7%, scleractinians: 13.6 ± 10.2%, serpulids: 8.8 ± 7.6%, bryozoans: 2.3 ± 1.2%, encrusting algae: 1.3% ± 1.2% (Fig. 8c). The analysis of images showed that the bioconstructions were characterized by marked porosity (73.2 ± 3.3%) that was due to spaces within and between individuals and small-scale porosity related to bioerosion (Figs. 8 and 9). The boring sponges occurred in all sampled material at both sites, where they heavily bioeroded carbonate structures, showing a clear decreasing gradient of perforation from the oldest to the youngest parts of the bioconstruction (Fig. 9).

### Taxonomic accounts and autecological features

**Figure 10:**
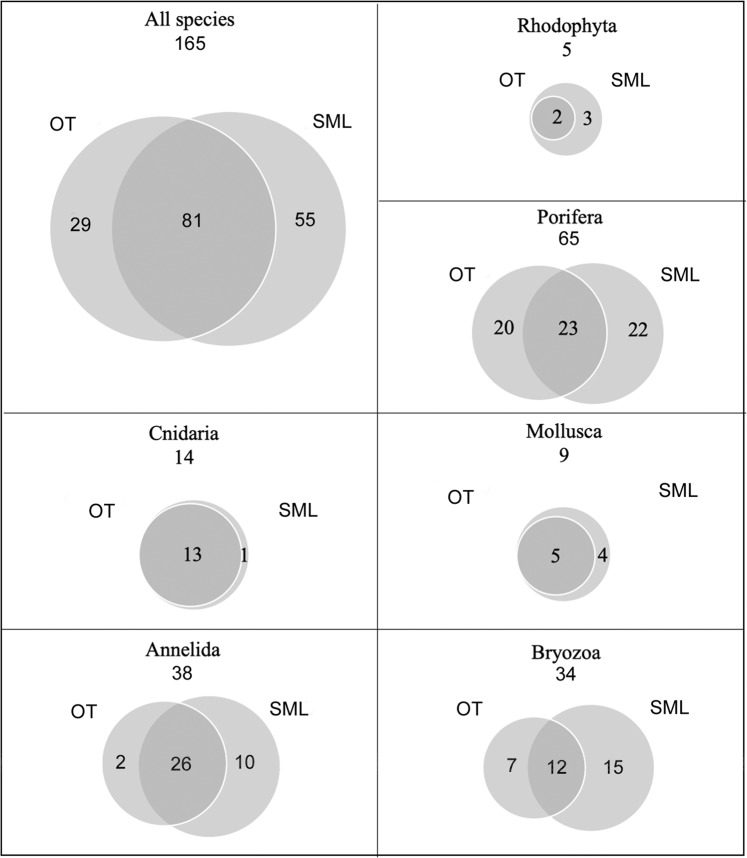
Venn diagrams showing relationships among the sampling areas at Otranto (OT) and Santa Maria di Leuca (SML) in terms of total species richness and main structuring taxa. The numbers in the overlapping areas indicate the shared taxa, those in the external areas indicate the exclusive taxa.

Analysis of the biological samples revealed the presence of a total of 165 structuring taxa (153 identified to the species level), 110 of which were detected at OT and 136 at SML (Supplementary Table S1), and 81 (49%) were shared by both sites. Overall, the phylum Porifera had the greatest species richness (65 taxa), followed by Annelida (38 taxa) and Bryozoa (34 taxa), while algae were present to a lesser extent (5 taxa). The patterns of species of the different taxa showed total overlap between the two study sites for algae, cnidarians and bivalves, with SML hosting all the taxa recorded at OT plus some exclusive ones. In contrast, sponges, annelids and bryozoans diverged in terms of species composition (Fig. 10).

### Algae

A total of 5 species of the class Florideophyceae were identified (Supplementary Table S1). Three of them are non-geniculated encrusting forms: *Titanoderma pustulatum* (J.V. Lamouroux) Nägeli, 1858 and *Lithophyllum stictiforme* (J.E. Areschoug) Hauck, 1877 belonging to the order Corallinales, and *Mesophyllum alternans* (Foslie) Cabioch & M.L. Mendoza, 1998 belonging to the order Hapalidiales. *Jania* sp. and *Amphiroa* sp. are geniculated species belonging to the order Corallinales. *T. pustulatum* and *M. alternans* are the only species that were detected in both study areas. Coralline species showed a patchy pattern in both study areas, where they were represented by small thalli, with a maximum surface covering of a few square centimetres. Encrusting species were attached to tiny rocks, scleractinians and mollusc shells. Geniculated species were attached to encrusting coralline species or other Rhodophyta species.

### Porifera

**Porifera** encompassed 65 taxa, with 61 identified to the species level, as follows: 4 species of Homoscleromorpha (1 order, 2 families, 4 genera) and 61 of Demospongiae (3 subclasses, 13 orders, 30 families, 41 genera) (Supplementary Table S1). The order Dictyoceratida was most represented, with 4 families, 6 genera and 11 species. Poecilosclerida was represented by 4 families, 5 genera and 8 species. Finally, Axinellida, Haplosclerida, and Tetractinellida were other representative orders. Most of the sponge taxa recorded show an Atlanto-Mediterranean distribution, and 12 taxa are currently reported only in the Mediterranean Sea^28^.

The number of sponge taxa found at the study sites was 43 at OT and 45 at SML, 23 of which were shared, with a high number of exclusive species (20 and 22, respectively) (Fig. 10).

In general, encrusting forms prevailed at both sites, and the few massive and erect species, when present, were of small size. Among the encrusting forms, *Dendroxea lenis* (Topsent, 1892) showed the highest frequency (Fig. 6a), *Axinella verrucosa* (Esper, 1794) was the most frequent sponge among erect species (Fig. 6), and massive species were only sporadically detected. Seven species were boring sponges, but only one of them (*Siphonodictyon infestum* (Johnson, 1889)) was widely represented in samples from both sites; this species is able to produce large boring chambers in the shell walls of *N. cochlear* (Fig. 4i,j).

### Cnidaria

Fourteen anthozoan taxa belonging to the orders Alcyonacea (3 families and 4 genera), Scleractinia (4 families, 9 genera) and Zoantharia (1 family, 1 genus) were detected in both study areas (Supplementary Table S1). Most of the species recorded have an Atlanto-Mediterranean distribution, with the exception of *Eunicella cavolini* (von Koch, 1887) and *Parazoanthus axinellae* (Schmidt, 1862), whose current known distribution is limited to the Mediterranean^29^.

The two study areas showed high similarity in terms of composition of the Anthozoa. Indeed, with the exception of *Alcyonium coralloides* (Pallas, 1766), detected at SML only, all the species were found at both sites (Fig. 10).

The order Scleractinia was the most represented in terms of species richness and frequency of occurrence. Scleractinians colonized the shells of *N. cochlear* or settled inside and between the valves of the empty shells throughout the bioconstruction. *Hoplangia durotrix* Gosse, 1860 and *Caryophyllia (Caryophyllia) inornata* (Duncan, 1878) were particularly abundant, scattered throughout the framework. *Cladopsammia rolandi* Lacaze-Duthiers, 1897 and *Leptopsammia pruvoti* Lacaze-Duthiers, 1897 formed large facies mainly in shaded regions of the bioconstruction (Fig. 4d,e). Finally, *C. (C.) smithii* Stokes & Broderip, 1828, *Monomyces pygmaea* (Risso, 1826) and *Stenocyathus vermiformis* (Pourtalès, 1868) were more rarely recorded.

Among the alcyonaceans*, Paramuricea clavata* (Risso, 1826) showed the highest density, with large colonies reaching 50 cm in fan diameter (Fig. 4h).

### Mollusca

The living mollusc fauna sampled at OT and SML was represented by 9 species belonging to the classes Gastropoda (5) and Bivalvia (4) (Supplementary Table S1). Among the Gastropoda, 4 species belonged to the order Littorinimorpha and 1 to the order Lepetellida. With regard to bivalves, *N. cochlear* belonged to the order Ostreida and to the family Gryphaeidae, and the other 3 species belonged to the orders Galeommatida (1 species) and Adapedonta (2 species). All the mollusc species have an Atlanto-Mediterranean distribution, with the exception of the gastropods *Alvania carinata* (da Costa, 1778) and *Sandalia triticea* (Lamarck, 1810), which have a distribution limited to the eastern Mediterranean basin (Horton et al., 2019). The two study areas shared 55% (2 Gastropoda and 3 Bivalvia) of the mollusc fauna, while the remaining 45% (4 species) was exclusively found at SML (Fig. 10). In general, the mollusc fauna was characterized by low abundance values, with the exception of *N. cochlear*, the main builder of the bioconstructions. Living specimens of *N. cochlear* formed clusters of a few individuals scattered on the upper part of the calcareous framework, while most of the bioconstruction was composed of dead specimens. The abundance of living specimens of *N. cochlear* calculated for 300 ml of the bioconstruction varied from 1.7 ± 0.7 to 9.1 ± 2.4 at OT and SML, respectively. In particular, the living specimens detected in the OT samples were mainly represented by juveniles (1.3 ± 0.2%) with the length of the main valve measuring approximately 1 cm.

Regarding the other mollusc species, most were recorded inside and between the valves of dead specimens of *N. cochlear*, although *Vermetus triquetrus* Bivona-Bernardi, 1832 colonized the exposed surface of the bioconstruction, covering some of the *Neopycnodonte* valves. The gastropod *Pseudosimnia carnea* (Poiret, 1789) is locally abundant and was mainly found to be associated with red coral colonies, on which it is parasitic.

### Annelida

Thirty-eight species of Serpulidae, with 29 Serpulinae and 9 Spirorbinae, were recorded (Supplementary Table S1). Almost all species (36) were collected at SML, and 28 species were found at OT, with 26 shared between the two sites. Most species were found with few individuals, while *Vermiliopsis infundibulum* (Philippi, 1844)*, Semivermilia crenata* (O.G. Costa, 1861)*, Filogranula annulata* (O.G. Costa, 1861) and *Semivermilia pomatostegoides* (Zibrowius, 1969), together with some spirorbids, such as *Protolaeospira (Protolaeospira) striata* (Quiévreux, 1963) and *Pileolaria militaris* Claparède, 1870, were particularly abundant. Such polychaetes have a Mediterranean and north-eastern Atlantic distribution, and some of them are cosmopolitan, e.g., *V. infundibulum, S. vermicularis, Josephella marenzelleri* Caullery & Mesnil, 1896*, P. militaris, Neodexiospira pseudocorrugata* (Bush, 1905), and *Janua heterostropha* (Montagu, 1803); 6 species are endemic to the Mediterranean, i.e., *Placostegus crystallinus* (non Scacchi, 1836) *sensu* Zibrowius, 1968*, S. pomatostegoides, V. monodiscus* Zibrowius, 1968, and *Spirobranchus lima* (Grube, 1862), and 3 species, i.e., *Serpula cavernicola* Fassari & Mollica, 1991*, S. annularis* Dillwyn, 1817 and *Nidificaria clavus* (Harris, 1968), only occur in the Mediterranean Sea, Gibraltar area and Canary Islands. The polychaete species exhibit a high level of adaptive radiation and can be ascribed to different ecological groups^30–32^: meso-infralittoral shelf species, e.g., *Spirobranchus polytrema* (Philippi, 1844)*, S. triqueter (*Linnaeus, 1758) and *Janua heterostropha* (Montagu, 1803); characteristic coralligenous species, e.g., *S. crenata* and *Vinearia koehleri* (Caullery & Mesnil, 1897); detrital continental shelf species, e.g., *S. cribrata* (O.G. Costa, 1861) and *Spirorbis (Spirorbis) cuneatus* Gee, 1964; deep-water and bathyal species, e.g., *V. monodiscus, Serpula israelitica* Amoureux, 1977, and *Filogranula gracilis* Langerhans, 1884; and cave species, e.g., *S. cavernicola* and *F. annulata*.

### Bryozoa

Thirty-four taxa of bryozoans were identified: 30 belonging to the class Gymnolaemata, order Ctenostomatida, and 4 belonging to the class Stenolaemata, order Cyclostomatida (the latter were not identified to the species level) (Supplementary Table S1). The bioconstructions at SML showed a higher species richness (27 taxa) with respect to that at OT (19 taxa). Twelve taxa were shared between the two sites, while the exclusive species accounted for 21% at OT and 44% at SML (Fig. 10). Most of the bryozoan species are distributed in the north-eastern Atlantic Ocean and largely in the Mediterranean Sea, but some of them, e.g., *Schizoporella mutabilis* Calvet, 1927*, Schizoretepora serratimargo* (Hincks, 1886)*, Rhynchozoon* sp., and *Pentapora fascialis* (Pallas, 1766), are endemic to the Mediterranean.

Many taxa were found living close to one another. Most of them exhibited both thick (11 taxa) and thin (7 taxa) encrusting habitus, others were present in petraliform and celleporiform colonies (3 species), and 7 taxa were found to form erect colonies. The encrusting species of the genus *Puellina* were the major occupiers of the substrate, together with *Schizomavella* and *Schizoporella* spp., which developed sheets that extensively covered the surface of the bioconstruction. Moreover, encrusting bryozoans grew epibiotically on serpulid tubes and on other bryozoan colonies. Bryozoans of the genera *Crassimarginatella* and *Beania*, with petraliform colonies, and those of the genera *Rhynchozoon* and *Turbicellepora*, with celleporiform colonies, populated interstices, cavities and crevices of the bioconstruction. The few erect bryozoans mainly colonized the outer edges of *Neopycnodonte* shells with both rigid (*Myriapora truncata* (Pallas, 1766) and *Crisia* sp.) and flexible *(Bugula gautieri* Ryland, 1962) colonies.

## Discussion

During recent years, there has been increasing interest in Mediterranean circalittoral and bathyal communities, mostly due to technological improvements, which have provided increased investigation accessibility to the deepest benthic areas. These explorations have emphasized the high species richness and diversity of the benthic assemblages that thrive in such ecosystems^33,34^ and the notable role of engineer animal species in building 3D animal forests^35^. Most such studies have stressed the role of scleractinians as main reef-building organisms^5–7^, highlighting the paramount ecological role of such calcifying bioconstructors^36^. At Mediterranean scale, literature data on marine gastropod bioconstructions have mainly focused on shallow waters, where large vermetid reefs are known from the Late Miocene and from off Israel^37–39^. In contrast, very limited information is available about biogenic formations built by bivalves on circalittoral and bathyal seabeds because most of the literature mainly reported distributional data rather than providing a fine-scale description of such formations.

In the bathyal environment, the few existing data concern the unique coral-bivalve biotope, where the deep-sea oyster *Neopycnodonte zibrowii* Gofas, Salas and Taviani, 2009 is described as a notable builder species^40–42^. In mesophotic environments, the congeneric *N. cochlear* was reported to be able to make biogenic formations scattered over both soft and hard substrates^24^ or build thick bioconstructions on the walls of submerged karst dolines along the northern Apulian coast^16^. In addition, *N. cochlear* was one of the secondary bioconstructors in the coral reef recently described on the northern Apulian coast^5^.

The present study describes at a fine scale and with a multidisciplinary approach the massive bioconstructions built by *N. cochlear*, including their local distribution, morphological framework and structuring taxa. The bioconstructions recorded off the southern Italian coast (northern Ionian Sea) resulted unnoticed until now despite past investigations carried out in the same geographic area^18,43^. The novelty of the present paper is the description of large and thick biogenic formations never observed before for this species. At both study sites, the bioconstructions showed a wide extension and appeared as complex frameworks entirely composed of a large number of living and dead specimens of *N. cochlear* associated with numerous other taxa with accessory structural function, helping to increase habitat heterogeneity.

In comparison with Mediterranean coralligenous reefs *sensu stricto*^2,4^ and the recently described mesophotic coral reef^5^, the *Neopycnodonte* bioconstruction showed peculiar features, since it lacked the major contributions of encrusting coralline algae and scleractinians as reef builders, respectively. The bioconstruction built by *N. cochlear* was very complex and diversified in the associated community of structuring organisms. It hosted a large number of benthic species attributable to different ecological groups occurring in different microhabitats of the bioconstruction.

Overall, the main structuring species were represented by invertebrate suspension feeders, suggesting the high trophic availability of the surrounding waters. The calcareous framework resulted from the stratification of different generations of benthic invertebrates, with the highest contribution of *N. cochlear*. Conversely, algae were poorly represented both in terms of frequency of occurrence and species richness. *Mesophyllum alternans* and *Titanoderma pustulatum*, which compose a large part of coralligenous bioconstructions^2^, although observed in both study areas, showed a patchy distribution and were represented by small-sized thalli. The scarce presence and low diversity of coralline algae, usually well represented in this bathymetric range, might be explained by the high sediment deposition observed in both study areas. Indeed, high sedimentation rates, together with water movement and pH, are usually considered to be the main factors limiting the growth of coralline algae^2,44,45^. Scleractinians showed a dominant role among secondary structuring taxa, colonizing the valves of dead *N. cochlear* specimens and becoming embedded within the calcareous frame. The alcyonaceans played a predominant role as 3D habitat makers, in accordance with the literature which describes such arborescent invertebrates as being able to form complex animal forests^35^. Serpulid polychaetes as well exhibited a notable role in increasing habitat heterogeneity, with a large number of tubes, mostly represented by species typical of shallow and detrital bottoms, being cemented to the outer portions of the bioconstruction. Species characteristic of deep-water biotopes as well as of cryptic microhabitats and caves preferentially colonized *Neopycnodonte* valves and the interstices of the structure. Most of the spirorbids showed a pioneering role, as their tubes settled on bare substrate, such as the external edges of living *Neopycnodonte* valves and their smooth inner parts. In addition to this colonization pattern, in accordance with their small dimensions and often-wrapped tubes, spirorbids particularly adapted themselves to cryptic interstices and dark crevices of the bioconstruction^31^. Within the bioconstruction, it was also noteworthy that the spirorbid-bryozoan interaction was exhibited by encrusting bryozoans’ extensive cover on most of the spirorbids’ tubes. Bryozoans settled as epibionts on other organisms and offered their colonies as a suitable surface for subsequent colonization. Most of them showed unilaminar encrusting growth and were typical of deep-water habitats subjected to low light intensity^46–49^. In particular, *Schizomavella* and *Schizoporella* species mainly played the role of binders, forming sheets that covered large portions of the bioconstruction. Poriferans were dominant in terms of number of taxa. They were mainly represented by encrusting forms, with a scarce contribution of massive and erect specimens. Overall, their role as 3D habitat makers appeared to be negligible, while their function as substrate binders was remarkable. On the other hand, their action as substrate eroders appeared to be very important because of the abundance of boring species throughout the bioconstruction. In particular, *Siphonodictyon infestum* was always present on the shells of dead specimens of *N. cochlear*, appearing to be increasingly pervasive towards the deeper layers of the bioconstruction.

Overall, the benthic assemblage associated with the *Neopycnodonte* bioconstruction showed a certain degree of variability between the two study areas, with differences depending on the taxonomic group. The overlap of species was approximately 50% of the total, and SML had a greater number of exclusive species than OT. Differences were negligible for cnidarians, molluscs and algae, while they were greater for annelids, poriferans and bryozoans, thus suggesting different ecological conditions between the two sites. In this regard, an important role could be played by the strong currents occurring at SML, where waters of the Ionian Sea and Canale d’Otranto meet, generating water turbulence that also affects the deeper portions of the seabed^50,51^, thus determining a different food supply in the two areas.

Similar to what has already been noted for coralligenous and other Mediterranean bioconstructions^5,6,20,52^, the *Neopycnodonte* bioconstruction enhances habitat heterogeneity and promotes biodiversity, thus supplying ecosystem services for human society^27^. For this reason, biogenic structures formed by the mollusc habitat-forming species *N. cochlear* and *N. zibrowii* are already included on the list of Marine Habitat Types for the Selection of Sites in the National Inventories of Natural Sites of Conservation Interest in the Mediterranean Sea^53^. In particular, *N. cochlear* is included in the section of circalittoral rocky habitats and *N. zibrowii* in the bathyal rocky habitats section^53^. Furthermore, because of their sensitivity to different anthropic impacts, such bioconstructions are classified as Vulnerable Marine Ecosystems according to the WGVME (2017) of the General Fisheries Commission for the Mediterranean.

We recognize that animal-dominated biogenic formations would have larger extensions in the south Adriatic twilight zone, and a larger, similar bioconstruction is currently under investigation in the central Adriatic Sea (unpublished data from the same authors). In addition, we are aware of the need for better knowledge of both the occurrence and extent of such vulnerable habitats and their main biological aspects. These latter include the functional roles and life history traits of the species, to monitor their environmental status, assess possible adverse impacts and establish sustainable management strategies. Finally, the need to improve the knowledge on Mediterranean mesophotic bioconstructions seems to have become even more crucial in the light of the recent finding of remarkable scleractinian bioconstructions in the same bathymetric belt^5^. This highlights the need to better clarify the identity of mesophotic bioconstructions in the Mediterranean basin, until now numbered in the great mosaic of coralligenous formations, even though often structurally different from the coralligenous *sensu stricto*^2,4^.

## Material and methods

### Marine geology procedures

Vessel positioning was carried out with the Differential Global Positioning System (DGPS) and a TRIMBLE SPS551 (USA) by means of the navigation software TELEDYNE(DK) PDS2000. Two morpho-bathymetric maps of approximately 0.10 and 0.25 km^2^ area of the Otranto (OT) and Santa Maria di Leuca (SML) shelf, respectively (Figs. 2 and 3), were obtained by using a KLEIN 3000 Side-Scan Sonar (SSS) (100–500 KHz operating frequencies) and by processing the data with CARIS SIPS. The results were input into a geographic information system (GIS) project (ESRI ArcView 10.2; projection UTM33N-WGS84). A multibeam survey (R2SONIC – USA 2022 with a 450 kHz-frequency pulse integrated with an I2NS attitude-direction-position system) was carried out to obtain a high-resolution digital elevation model (DEM) of the seafloor that was useful for recognizing the main morphological features associated with the bioconstructions. High-resolution SSS greyscale images (0.2 m pixel raw dataset and processed mosaicked image) were used for the identification of the largest bioconstructions. In the GIS environment, detailed mapping based on the geophysical features of the bioconstructions was executed with geo-referenced images; geophysical-based polygons were used to inform diving and sampling procedures.

### Video acquisition and sample collection

To validate the interpretation of the mosaicked sonograms, describe the “architecture” of the bioconstructions and characterize the associated epibenthic assemblages in terms of structuring taxa, 6 georeferenced underwater video transects were performed in each study area. Specifically, the video transects were carried out vertically on rocky walls at a depth ranging from 39 to 64 m at OT and from 27 to 70 m at SML (Figs. 2f and 3d) by technical divers equipped with high-definition video cameras (Sony PMW-EX1 and Sony Alpha 7III), high-performance LED strobe illuminators (EasyDive, 13,000 lumens) and 3 laser beams providing a 10 cm-scale for measuring sampling areas on the substrate and quantitative data of the community. Scuba dives were carried out at a minimum distance of 50 m from one another, with locations selected according to the mosaicked sonograms or where the signal that was returned according to the geophysical survey was not sufficient to exactly define the type of biological association that was present (*Neopycnodonte vs* coralligenous bioconstructions *sensu stricto* or soft-bottom communities). Additional dives were performed to collect samples for the fine-scale description of the bioconstructions. For this purpose, in each study area, 3 biological samples (each approximately 3 l in volume) were collected for taxonomical analysis in different areas and depth intervals of the bioconstruction. Further samples (approximately 3 l in volume) were collected from the same areas to describe the structural organization of the bioconstruction.

### Taxonomic and structural analysis

The complete list of species contributing to the bioconstruction (Supplementary Table S1) was obtained by the examination of video images and the analysis of samples. Video images were evaluated using VLC media player free software. All megabenthic organisms observed in the images within a visual field of 50 cm were recorded to the lowest possible taxon.

Biological material was sorted, and all specimens were fixed in a 5% formaldehyde solution with seawater and then stored in a 70% ethanol solution. To identify the sampled taxa, an appropriate procedure of preparation and identification of each taxonomic category was applied. The collected biological material was identified to the lowest possible taxonomic level. The taxonomic nomenclature referred to the World Register of Marine Species (WORMS)^29^.

The contribution of each structuring taxon to the bioconstruction was evaluated by analysing 20 video frames obtained from each video transect recorded on the *Neopycnodonte* bioconstruction (Fig. 2f: Dive 01, Dive 02, Dive 03, Dive 05, Dive 06 for OT and Fig. 3d: Dive 01, Dive 02, Dive 03, Dive 04, Dive 05, Dive 06 for SML), for a total of 100 frames for OT and 120 frames for SML. The frames were extracted using the freely available DVDVideoSoft Free Studio, and image analysis was performed using ImageJ software. The relative frequencies of the main structuring taxa were calculated on the basis of the number of frames for each transect in which the taxon occurred. The covering values were calculated by superimposing a grid of 9 subsquares onto each image and counting the number of subsquares within which each taxon was present. The living *Neopycnodonte* specimens were counted in ten samples from the bioconstruction of 300 ml each. For each live specimen, the major axis of the upper valve was measured.

The small-scale 3D structure of approximately 3 l samples of the bioconstruction was described. The samples were washed in the laboratory with distilled water and dried in an oven. The original framework of the bioconstruction was preserved, saturating its pores with a low-viscosity epoxy resin. An impregnation procedure was specifically developed for these high-porosity samples, enveloping them with a plastic coat using a large bell jar vacuum and 6 cycles of resin saturation. After this procedure, the samples were ready to be cut into slices (6 slices of various cm in thickness for every sample) with a circular saw to allow the direct observation of the architecture of the bioconstruction without deforming it. High-resolution images of the resin slices were obtained with a scanner. Image analyses were carried out using ImageJ software to describe the general framework of the bioconstruction, to measure the relative abundance of builder taxa by calculating their surface fraction and to evaluate the porosity of the bioconstruction.

## Supplementary information

Supplementary information.

Supplementary video 1.

Supplementary video 2.

Supplementary video 3.

Supplementary video 4.
