# Supplementary material for: Massive bioconstructions built by Neopycnodonte cochlear (Mollusca, Bivalvia) in a mesophotic environment in the central Mediterranean Sea
Source: Sci Rep. 2020 Apr 14;10:6337. doi: 10.1038/s41598-020-63241-y (PMC7156399; doi:10.1038/s41598-020-63241-y)
Supplement: Supplementary file 1 — Supplementary information. [file 41598_2020_63241_MOESM1_ESM.pdf]

Supplementary Information for:

“MASSIVE BIOCONSTRUCTIONS BUILT BY *NEOPYCNODONTE COCHLEAR* (MOLLUSCA, BIVALVIA) IN A MESOPHOTIC ENVIRONMENT IN THE CENTRAL MEDITERRANEAN SEA”

Frine Cardone, Giuseppe Corriero, Caterina Longo, Maria Mercurio, Senem Onen Tarantini, Maria Flavia Gravina, Stefania Lisco, Massimo Moretti, Francesco De Giosa, Adriana Giangrande, Carlotta Nonnis Marzano, Cataldo Pierri

SUPPLEMENTARY TABLE AND FIGURE CAPTIONS

SUPPLEMENTARY VIDEO CAPTIONS

The supplementary videos 1 and 2 were recorded by technical divers on the *Neopycnodonte* bioconstructions off Otranto and Santa Maria di Leuca at a depth ranging from 40 to 70 m. The supplementary videos 3 and 4 are animations obtained by digital elevation models of the seafloor.

Supplementary Video *Neopycnodonte* bioconstruction 1

Video recorded on a vertical cliff off Otranto. In this video it is possible to observe the globose formations edified by *Neopycnodonte cochlear*, protruding perpendicularly from the cliff. The bioconstruction is plentifully populated by red coral, whose colonies aggregate especially below the biogenic formations built by *Neopycnodonte*. Large encrustations of the demosponge *Dendroxea lenis* (grey) and abundant plaques of the bryozoans *Schizomavella* spp. are also visible.

Supplementary Video *Neopycnodonte* bioconstruction 2

Video recorded on a vertical cliff off Santa Maria di Leuca. In this video it is possible to observe the thick pinnacles edified by *Neopycnodonte cochlear*, protruding perpendicularly from the cliff. The structural complexity of the bioconstructions and their marked thickness are easily recognizable. The framework is populated by large colonies of *Paramuricea clavata*. Red coral shows a patchy distribution mainly thriving below the pinnacles, where it is also easy to observe the demosponge *Aplysina cavernicola*.

Supplementary Video *Neopycnodonte* bioconstruction 3

A digital elevation model of the seafloor derived from the multibeam survey carried out at Otranto. The seafloor geometry is dominated by the presence of a slope that runs parallel to the coast connecting a large coastal flat area to deeper sectors. The animation shows the areas where the *Neopycnodonte* bioconstruction has been detected.

Supplementary Video *Neopycnodonte* bioconstruction 4

A digital elevation model of the seafloor derived from the multibeam survey carried out at Santa Maria di Leuca. The seafloor geometry is dominated by the presence of a slope that runs along an ENE-WSW direction, transversally to the coastline. The slope is locally steep and shows irregular morphology. Flat and shallow sectors pass laterally to a sub-vertical cliff where the *Neopycnodonte* bioconstruction has been intercepted.

Table S1. List of the structuring benthic taxa recorded in the Otranto and Santa Maria di Leuca *Neopycnodonte.* bioconstructions.

Phylum	Class	Order	Family	Species	OT	SML
RHODOPHYTA	FLORIDEOPHYCEAE	**Hapalidiales**	**Mesophyllaceae**	*Mesophyllum alternans* (Foslie) Cabioch & M.L.Mendoza, 1998	*	*
		**Corallinales**	**Corallinaceae**	*Jania* sp.		*
			**Lithophyllaceae**	*Amphiroa* sp.		*
			**Lithophyllaceae**	*Titanoderma pustulatum* (J.V.Lamouroux) Nägeli, 1858	*	*
			**Lithophyllaceae**	*Lithophyllum stictiforme* (J.E. Areschoug) Hauck, 1877		*
PORIFERA	HOMOSCLEROMORPHA	**Homosclerophorida**	**Oscarellidae**	*Oscarella lobularis* (Schmidt, 1862)	*	
			**Plakinidae**	*Corticium candelabrum* Schmidt, 1862		*
				Plakina trilopha Schulze, 1880		*
				Plakortis simplex Schulze, 1880	*	*
	DEMOSPONGIAE	**Tetractinellida**	**Ancorinidae**	*Dercitus* (*Stoeba*) *plicatus* (Schmidt, 1868)	*	
				Dercitus sp.	*	
				Jaspis johnstonii (Schmidt, 1862)	*	*
			**Geodiidae**	*Geodia conchilega* Schmidt, 1862	*	*
			**Pachastrellidae**	*Triptolemma simplex* (Sarà, 1959)	*	
			**Thoosidae**	*Delectona ciconiae* Bavestrello *et al.*, 1996		*
				Thoosa armata Topsent, 1888		*
		**Clionaida**	**Clionaidae**	*Cliona janitrix* Topsent, 1932	*	*
				Cliona viridis Schmidt, 1862		*
				Spiroxya heteroclita Topsent, 1896		*
				Spiroxya sarai (Melone, 1965)		*
			**Spirastrellidae**	*Diplastrella bistellata* (Schmidt, 1862)		*
		**Chondrosiida**	**Chondrosiidae**	*Chondrosia reniformis* Nardo, 1847	*	*
		**Suberitida**	**Suberitidae**	*Protosuberites denhartogi* van Soest & de Kluijver, 2003		*
				Suberites syringella (Schmidt, 1868)	*	
				Terpios gelatinosus (Bowerbank, 1866)	*	*
			**Halichondriidae**	*Haliclona poecillastroides* (Vacelet, 1969)		*
		**Bubarida**	**Bubaridae**	*Bubaris carcisis* Vacelet, 1969		*
				Bubaris vermiculata (Bowerbank, 1866)		*
			**Dictyonellidae**	*Acanthella acuta* Schmidt, 1862	*	*
				Dictyonella incisa (Schmidt, 1880)	*	
		**Axinellida**	**Axinellidae**	*Axinella cannabina* (Esper, 1794)		*
				Axnella damicornis (Esper, 1794)	*	*
				Axinella polypoides Schmidt, 1862		*
				Axinella verrucosa (Esper, 1794)	*	*
			**Raspailiidae**	*Eurypon denisae* Vacelet, 1969	*	
				Eurypon viride (Topsent, 1889)	*	
				Raspaciona aculeata (Johnston, 1842)	*	
		**Biemnida**	**Rhabderemiidae**	*Rhabderemia indica* Dendy, 1905	*	
		**Poecilosclerida**	**Crambeidae**	*Crambe crambe* (Schmidt, 1862)	*	*
			**Hymedesmiidae**	*Hemimycale columella* (Bowerbank, 1874)	*	
				Phorbas tenacior (Topsent, 1925)	*	*
			**Microcionidae**	*Clathria* (*Clathria*) *toxistricta* (Topsent, 1925)		*
				Clathria (*Microciona*) *gradalis* Topsent, 1925	*	
				Clathria (*Microciona*) *spinarcus* (Carter & Hope, 1889)		*
				Clathria sp.	*	
			**Mycalidae**	*Mycale* (*Mycale*) *lingua* (Bowerbank, 1866)		*
		**Agelasida**	**Agelasidae**	*Agelas oroides* (Schmidt, 1864)	*	*
			**Hymerhabdiidae**	*Prosuberites longispinus* Topsent, 1893	*	
		**Haplosclerida**	**Chalinidae**	*Dendroxea lenis* (Topsent, 1892)	*	*
				Haliclona (*Gellius*) *flagellifera* (Ridley & Dendy, 1886)		*
				Haliclona (*Halichoclona*) *fulva* (Topsent, 1893)	*	
				Haliclona (*Reniera*) *mediterranea* Griessinger, 1971	*	*
				Haliclona (*Reniera*) sp.		*
			**Petrosiidae**	*Petrosia* (*Petrosia*) *ficiformis* (Poiret, 1789)	*	
			**Phloeodictyidae**	*Siphonodictyon infestum* (Johnson, 1889)	*	*
		**Dendroceratida**	**Dictyodendrillidae**	*Spongionella gracilis* (Vosmaer, 1883)	*	
		**Dictyoceratida**	**Dysideidae**	*Dysidea avara* (Schmidt, 1862)	*	*
				Dysidea fragilis (Montagu, 1818)		*
				Dysidea sp.	*	
				Pleraplysilla spinifera (Schulze, 1878)	*	*
			**Irciniidae**	*Ircinia dendroides* (Schmidt, 1862)	*	
				Ircinia sp.		*
				Ircinia variabilis (Schmidt, 1862)	*	*
				Sarcotragus foetidus Schmidt, 1862	*	*
				Sarcotragus spinosulus Schmidt, 1862	*	*
			**Spongiidae**	*Spongia* (*Spongia*) *nitens* (Schmidt, 1862)	*	
			**Thorectidae**	*Scalarispongia scalaris* (Schmidt, 1862)	*	*
		**Verongiida**	**Aplysinidae**	*Aplysina cavernicola* Vacelet, 1959	*	*
			**Ianthellidae**	*Hexadella* cf. *pruvoti* Topsent, 1896		*
				Hexadella racovitzai Topsent, 1896	*	*

CNIDARIA	ANTHOZOA	**Alcyonacea**	**Alcyoniidae**	*Alcyonium coralloides* (Pallas, 1766)		*
			**Coralliidae**	*Corallium rubrum* (Linnaeus, 1758)	*	*
			**Gorgoniidae**	*Eunicella cavolini* (von Koch, 1887)	*	*
			**Ple*auridae**	*Paramuricea clavata* (Risso, 1826)	*	*
		**Scleractinia**	**Caryophylliidae**	*Caryophyllia* (*Caryophyllia*) *inornata* (Duncan, 1878)	*	*
				Caryophyllia (*Caryophyllia*) *smithii* Stokes & Broderip, 1828	*	*
				Hoplangia durotrix Gosse, 1860	*	*
				Polycyathus muellerae (Abel, 1959)	*	*
				Phyllangia americana mouchezii (Lacaze-Duthiers, 1897)	*	*
			**Flabellidae**	*Monomyces pygmaea* (Risso, 1826)	*	*
			**Stenocyathidae**	*Stenocyathus vermiformis* (Pourtalès, 1868)	*	*
			**Dendrophylliidae**	*Cladopsammia rolandi* Lacaze-Duthiers, 1897	*	*
				Leptopsammia pruvoti Lacaze-Duthiers, 1897	*	*
		**Zoantharia**	**Parazoanthidae**	*Parazoanthus axinellae* (Schmidt, 1862)	*	*
MOLLUSCA	GASTROPODA	**Lepetellida**	**Fissurellidae**	*Emarginula octaviana* Coen,1939		*
		**Littorinimorpha**	**Rissoidae**	*Alvania carinata* (Da Costa, 1778)		*
			**Vermetidae**	*Vermetus triquetrus* Bivona Ant., 1832	*	*
			**Ovulidae**	*Sandalia triticea* (Lamarck, 1810)		*
			**Amathinidae**	*Clathrella clathrata* (Philippi, 1844)	*	*
	BIVALVIA	**Ostreida**	**Gryphaeidae**	*Neopycnodonte cochlear* (Poli, 1795)	*	*
		**Galeommatida**	**Lasaeidae**	*Kellia suborbicularis* (Montagu, 1803)	*	*
		**Adapedonta**	**Hiatellidae**	*Hiatella arctica* (Linnaeus, 1767)	*	*
				Hiatella rugosa (Linnaeus, 1767)		*
ANNELIDA	POLYCHAETA	**Sabellida**	**Serpulidae**	*Filogranula annulata* (O. G. Costa, 1861)	*	*
				Filogranula calyculata (O. G. Costa, 1861)	*	*
				Filogranula gracilis Langerhans, 1884		*
				Hydroides pseudouncinata Zibrowius, 1968	*	
				Janita fimbriata (Delle Chiaje, 1822)		*
				Josephella marenzelleri Caullery & Mesnil, 1896	*	*
				Metavermilia multicristata (Philippi, 1844)	*	*
				Placostegus crystallinus Zibrowius, 1968	*	*
				Placostegus tridentatus (Fabricius, 1779)	*	*
				Semivermilia agglutinata (Marenzeller, 1893)	*	*
				Semivermilia crenata (O. G. Costa, 1861)	*	*
				Semivermilia cribrata (O. G. Costa, 1861)	*	*
				Semivermilia pomatostegoides (Zibrowius, 1969)	*	*
				Serpula cavernicola Fassari & Mollica, 1991	*	*
				Serpula concharum Langerhans, 1880	*	*
				Serpula israelitica Amoureux, 1977		*
				Serpula lobiancoi Rioja, 1917	*	*
				Serpula vermicularis Linnaeus, 1767	*	*
				Spiraserpula massiliensis (Zibrowius, 1968)	*	*
				Spirobranchus triqueter (Linnaeus, 1758)	*	*
				Spirobranchus lima (Grube, 1862)		*
				Bathyvermilia eliasoni (Zibrowius, 1970)		*
				Spirobranchus polytrema (Philippi, 1844)		*
				Vermiliopsis infundibulum (Philippi, 1844)	*	*
				Vermiliopsis labiata (O. G. Costa, 1861)	*	*
				Vermiliopsis monodiscus Zibrowius, 1968		*
				Vermiliopsis striaticeps (Grube, 1862)	*	*
				Filograna implexa Berkeley, 1827	*	*
				Protula tubularia (Montagu, 1803)	*	*
				Janua heterostropha (Montagu, 1803)	*	*
				Neodexiospira pseudocorrugata (Bush, 1905)	*	*
				Nidificaria clavus (Harris, 1968)	*	
				Pileolaria militaris Claparède, 1870	*	*
				Pileolaria heteropoma (Zibrowius, 1968)		*
				Protolaeospira striata (Quiévreux, 1963)	*	*
				Spirorbis cuneatus Gee, 1964		*
				Spirorbis marioni Caullery &Mesnil, 1897	*	*
				Vinearia koehleri (Caullery & Mesnil, 1897)		*

BRYOZOA	STENOLAEMATA	**Cyclostomatida**		gen. 1 sp. 1	*	*
				gen. 2 sp. 1	*	*
				gen. 3 sp. 1		*
				Crisia sp.	*	*
	GYMNOLAEMATA	**Cheilostomatida**	**Calloporidae**	*Callopora dumerilii* (Audouin, 1826)		*
				Crassimarginatella crassimarginata (Hincks, 1880)		*
				Crassimarginatella maderensis (Waters, 1898)		*
			**Flustridae**	*Spiralaria gregaria* (Heller, 1867)		*
			**Bugulidae**	*Bugula gautieri* Ryland, 1962		*
			**Beaniidae**	*Beania magellanica* (Busk, 1852)	*	*
			**Cellariidae**	*Cellaria fistulosa* (Linnaeus, 1758)	*	
				Cellaria salicornioides Lamouroux, 1816	*	
			**Cribrilinidae**	*Cribrilaria setiformis* Harmelin & Aristegui, 1988		*
				Figularia figularis (Johnston, 1847)		*
				Glabrilaria pedunculata (Gautier, 1956)		*
				Puellina radiata (Moll, 1803)	*	*
				Puellina sp.		*
			**Escharellidae**	*Escharella rylandi* Geraci, 1974		*
			**Smittinidae**	*Smittina cervicornis* (Pallas, 1766)	*	
			**Bitectiporidae**	*Pentapora fascialis* (Pallas, 1766)	*	*
				Schizomavella auriculata (Hassall, 1842)	*	*
			**Schizoporellidae**	*Schizoporella dunkeri* (Reuss, 1848)	*	
				Schizoporella mutabilis Calvet, 1927		*
				Schizoporella magnifica Hincks, 1886	*	*
			**Myriaporidae**	*Myriapora truncata* (Pallas, 1766)	*	*
			**Cheiloporinidae**	*Hagiosynodos latus* (Busk, 1856)	*	
			**Lanceoporidae**	*Stephanotheca monoecensis* (Calvet, 1927)		*
				Stephanotheca watersi Reverter-Gil, Souto & Fernández-Pulpeiro, 2012		*
			**Escharinidae**	*Escharina vulgaris* (Moll, 1803)	*	*
			**Microporellidae**	*Microporella pseudomarsupiata* | (Aristegui, 1984)	*	
			**Celleporidae**	*Celleporina lucida* (Hincks, 1880)	*	
				Turbicellepora sp.	*	*
			**Phidoloporidae**	*Rhynchozoon pseudodigitatum* Zabala & Maluquer, 1988	*	*
				Schizoretepora serratimargo (Hincks, 1886)		*
